# Vulnerability of mineral-organic associations in the rhizosphere

**DOI:** 10.1038/s41467-025-61273-4

**Published:** 2025-07-01

**Authors:** Tobias Bölscher, Zoe G. Cardon, Mariela Garcia Arredondo, Stéphanie Grand, Gabriella Griffen, Rachel Hestrin, Josephine Imboden, Floriane Jamoteau, Emily M. Lacroix, Sherlynette Pérez Castro, Per Persson, William J. Riley, Marco Keiluweit

**Affiliations:** 1 Université Paris-Saclay, INRAE, AgroParisTech, UMR EcoSys, Palaiseau, France; 2 Department of Biology, Lund University, Lund, Sweden; 3 The Ecosystems Center, Marine Biological Laboratory, Woods Hole, MA USA; 4 Yale Center for Natural Carbon Capture, New Haven, CS USA; 5 Institute of Earth Surface Dynamics, University of Lausanne, Lausanne, Switzerland; 6 Stockbridge School of Agriculture, University of Massachusetts, Amherst, MA USA; 7 Centre for Environmental and Climate Science, Lund University, Lund, Sweden; 8 Climate and Ecosystem Sciences Division, Lawrence Berkeley National Laboratory, Berkeley, CA USA

**Keywords:** Carbon cycle, Ecosystem ecology, Microbial ecology, Element cycles

## Abstract

The majority of soil carbon (C) is stored in organic matter associated with reactive minerals. These mineral-organic associations (MOAs) inhibit microbial and enzymatic access to organic matter, suggesting that organic C within MOAs is resistant to decomposition. However, plant roots and rhizosphere microbes are known to transform minerals through dissolution and exchange reactions, implying that MOAs in the rhizosphere can be dynamic. Here we identify key drivers, mechanisms, and controls of MOA disruption in the rhizosphere and present a new conceptual framework for the vulnerability of soil C within MOAs. We introduce a vulnerability spectrum that highlights how MOAs characteristic of certain ecosystems are particularly susceptible to specific root-driven disruption mechanisms. This vulnerability spectrum provides a framework for critically assessing the importance of MOA disruption mechanisms at the ecosystem scale. Comprehensive representation of not only root-driven MOA formation, but also disruption, will improve model projections of soil C-climate feedbacks and guide the development of more effective soil C management strategies.

## Abstract (web summary)

Organic matter associated with reactive soil minerals is assumed to be the most persistent terrestrial carbon pool. This work introduces a novel mechanistic framework for the vulnerability of this vast carbon reservoir to disruption in the rhizosphere.

## Introduction

Plants allocate 40–60% of their photosynthetically fixed carbon (C) to their root systems and associated microbes^1,2^ via dead root cells, tissues, mucilage, and a cocktail of simple organic compounds—collectively known as rhizodeposits^3,4^. This rhizodeposition is a main source of soil organic matter (OM)^2,5,6^, the largest and most dynamic terrestrial C reservoir on Earth^7^. The proximity of roots to their surrounding mineral matrix is thought to facilitate the chemical association of rhizodeposits with reactive minerals^8–10^. These mineral-organic associations (MOAs) protect OM against microbial and enzymatic attack, potentially contributing to soil C storage for centuries to millennia^11,12^. Enhancing C storage through increased rhizodeposition and associated MOA formation has, consequently, become a climate mitigation strategy for policymakers, stakeholders, and scientists^13,14^. However, several lines of evidence suggest that rhizosphere processes effectively disrupt MOAs^15–20^, thereby enhancing microbial access to previously protected OM and diminishing soil C storage.

Plant roots and associated microbes have well-known nutrient mobilization strategies that transform minerals through dissolution and exchange reactions^21,22^. Particularly, the impact of small, soluble, and reactive organic compounds released by plants and associated microbes has received significant attention. The strong ligand oxalic acid, for instance, was found to directly attack minerals, liberating mineral-associated OM and rendering it accessible to microbes^16,18,23^. Even compounds considered nominally less reactive towards minerals, such as simple sugars, have been shown to disrupt MOAs through indirect, microbially or enzymatically-mediated mechanisms^17,18,20,24,25^. Although most of these studies rely on simple model compounds, they suggest that plant roots play a dual role by not only promoting the formation but also disrupting MOAs^19,20,25^.

Root-induced MOA disruption may not merely result from the release of simple reactive compounds, but rather arise from complex biogeochemical processes occurring within the dynamic rhizosphere environment (i.e., soil directly influenced by a plant root). MOAs are affected by changes in microbiome^26^, pore architecture^27,28^ and solute concentrations^3,4,21,29^ or shifts in pH and redox (E_h_) conditions^21,30–33^. Although such changes in biogeochemical conditions in the rhizosphere may trigger mineral transformations and liberation of mineral-associated organic matter, their impact on MOAs and C protected therein is rarely considered. Consequently, the potential of complex rhizosphere processes—beyond the effect of simple reactive compounds—to disrupt MOAs is missing from our current conceptual framework of the terrestrial C cycle. To predict how ecosystem carbon storage responds to shifts in environmental conditions (e.g., as induced by climate or land use change), a more comprehensive understanding of the intricate mechanisms driving MOA disruption in the rhizosphere, as well as associated microbial, edaphic, and plant factors that influence these processes, is needed.

This review aims to critically assess the vulnerability of MOAs to disruption in the rhizosphere. To achieve this, we examined known and potential controls, mechanisms, and environmental drivers of MOA disruption in the rhizosphere. Specifically, we reviewed and summarized (i) properties of MOAs that govern their inherent vulnerability to disruption, (ii) biogeochemical mechanisms by which roots can disrupt MOAs, and (iii) rhizosphere processes that trigger these disruption mechanisms. We then synthesized this information into a new conceptual framework for understanding MOA vulnerability in the rhizosphere. Finally, this framework serves as a basis for identifying future research priorities that explicitly account for the dynamic nature of MOAs. By addressing critical factors governing MOA disruption in the rhizosphere, we aim to illuminate their potential implications for the vast mineral-associated C reservoir and its vulnerability to shifts in environmental conditions.

### Terms and definitions

**Fig. 1:**
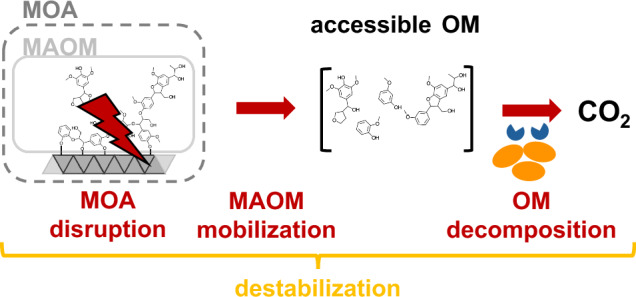
The disruption of mineral–organic associations (MOAs) causes the destabilization of mineral-associated organic matter (MAOM). Plants and associated microbes trigger *disruption* of MOAs through dissolution, desorption or depolymerization reactions that cleave bonds. Bond cleavage within MOAs enables the mobilization of previously inaccessible MAOM. Once mobilized, the resulting accessible organic matter (OM) can be subject to further microbial decomposition, either extracellularly or intracellularly, ultimately resulting in CO_2_ production. Thus, MAOM destabilization encompasses the full array of processes from MOA disruption to MAOM mobilization to OM decomposition. This review focuses on the effects of plant roots and associated microbes on MOA disruption and MAOM mobilization. Further, we define accessible OM as soluble as well as insoluble compounds accessible to microbial decomposition. We also note that disruption does not necessarily lead to decomposition of the newly accessible OM, but that temporarily accessible OM may form new MOAs and become MAOM again.

In-depth discussion of the dynamics of MOAs within the rhizosphere requires careful consideration and definition of the terminology. This review broadly defines root-induced processes as encompassing both those directly driven by roots as well as mediated by root-associated microbes in the rhizosphere. We define the *rhizosphere* as the soil surrounding roots that is biologically, chemically, or physically influenced by the living root^29,34,35^. Throughout this review, we use the term *mineral-organic associations* (MOAs) to describe the assemblage of mineral phases and OM, while *mineral-associated organic matter* (MAOM) denotes strictly the OM bound within these associations. We use the phrase *mineral phase* broadly, encompassing a range of inorganic phases across a spectrum of degrees of order and size: from single metal ions to amorphous metal phases to crystalline minerals. We define *disruption* mechanisms as any chemical reaction, enzymatically catalyzed or not, that breaks bonds between mineral phases and OM or between organic moieties within MAOM. *Mobilization* describes the resulting liberation of MAOM, creating nominally accessible OM with diverse properties (e.g., soluble vs. insoluble, large vs. small compounds) that is vulnerable to further decomposition but may also form new MOA and become MAOM again. *Decomposition* refers to both the extracellular *depolymerization* of larger OM into smaller molecules, enzymatically catalyzed or not, and the mineralization of assimilated molecules to CO_2_ via microbial respiration. MAOM *destabilization* is the combined (i.e., simultaneous or sequential) occurrence of MOA disruption, OM mobilization, and decomposition (Fig. 1).

### MOA characteristics in the rhizosphere

**Fig. 2:**
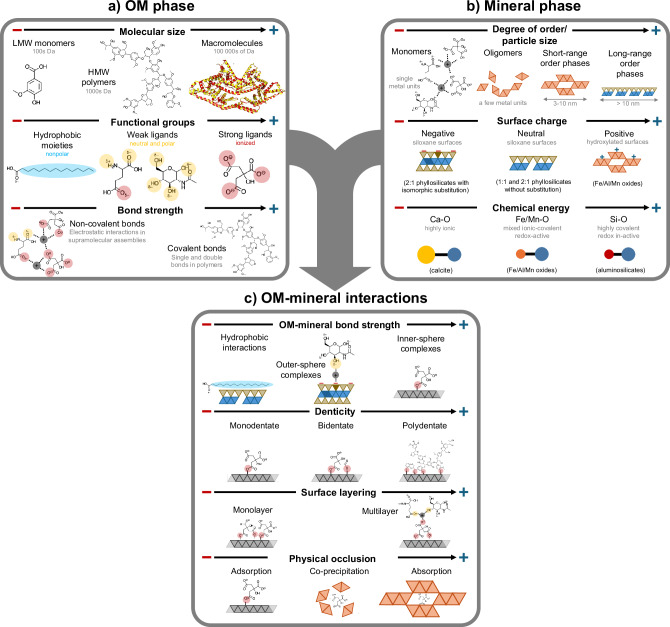
Physical-chemical properties of organic and mineral phases that govern the nature of organic matter–mineral interactions and, thus, the inherent vulnerability of resulting mineral-organic associations (MOAs) to disruption. MOA characteristics are differentiated based on the properties of **a** organic matter (OM) and **b** mineral phases. In both cases, the size, charge, and chemical stability are critical to define their reactivity. The individual properties of OM and mineral phases dictate the type of OM–mineral interactions. The chemical and physical nature of **c** OM–mineral interactions can be characterized by the type of chemical bond formed and the resulting bonding strength, denticity (i.e., the number of bonds formed), OM surface layering, and the degree of physical occlusion of OM in internal pores or coprecipitates. Arrows denote the direction in which given physical-chemical properties increase. Gray mineral phases depicted in panel **c** denote generic mineral surfaces. LMW and HMW denote low and high molecular weight.

The susceptibility of MOAs to disruption, and thus the potential vulnerability of MAOM to destabilization in the rhizosphere, depends on the physical-chemical properties of the organic and mineral components, as well as the interactions between them^36^. The physical-chemical characteristics of OM and mineral phases within MOA and emergent mineral-OM interactions are comprehensively reviewed elsewhere^36–38^. Here, we focus on specific characteristics of OM and mineral phases, and the resulting interactions that are relevant for assessing the vulnerability of MOA to disruption in the rhizosphere (Fig. 2). While the general characteristics of MOAs likely do not differ between bulk soil and the rhizosphere, the plant root creates a highly dynamic environment which makes the rhizosphere a potential hotspot of MOA disruption.

#### Organic matter characteristics

Once released into the soil environment, the affinity of rhizodeposits for minerals will depend on molecular size and functional groups, which both can be altered by microbial transformation of the rhizodeposits. Rhizodeposits range from low-molecular-weight metabolites (e.g., simple sugars and organic acids) to large macromolecules (e.g., proteins, phospholipids, or lignocellulose; Fig. 2a)^3,4^. The molecular size will affect the solubility of the compounds, but also the possible number of contact points with mineral surfaces^36^. Because functional groups determine polarity and charge of organic compounds^39,40^, their presence may dictate sorption mechanism and bonding strength by which rhizodeposits and their transformation products attach to mineral phases. Nonpolar, hydrophobic functional groups (e.g., lipids in root tissue) may interact differently with mineral phases than neutral and polar (e.g., amino sugars) or charged (e.g., organic acids) functional groups acting as strong or weak ligands. It is also important to consider organic-organic interactions within supramolecular and polymeric OM. OM composed of covalent polymers is bound by comparatively strong and rigid covalent bonds, while OM organized in supramolecular structures is held together by relatively weak non-covalent and reversible bonds^41^. Supramolecular and traditional covalent polymers may thus exhibit different sorption behavior on mineral phases. Consequently, by influencing the extent, type, and strength of possible OM–mineral interactions, OM molecular size, functional groups, and bond strength play a key role in the susceptibility of MOAs to disruption in the rhizosphere.

#### Mineral characteristics

The susceptibility of MOAs to disruption is influenced by the properties of the mineral reaction partner^39,40^. Key mineral phases—monomeric metal cations, imogolite-type minerals, Fe/Al/Mn oxides (e.g., ferrihydrite, goethite, gibbsite, and birnessite), and aluminum phyllosilicates (e.g., 2:1 or 1:1 layer silicates like smectite and kaolinite)—are defined by varying mineral crystallinity, charge, and bond strength (Fig. 2b). Crystallinity (or degree of order) describes the extent of repeating units in the mineral structure, ranging from monomeric units^42,43^ to oligomers^44,45^ to short-range and long-range order minerals^46–48^. Mineral phases with a low degree of order have smaller particle sizes and a greater specific surface area (e.g., 2-line ferrihydrite and amorphous Al(OH)_3_), which makes them more responsive to sorption of OM but also more susceptible to dissolution reactions than their more crystalline counterparts (e.g., goethite and gibbsite). That is why less ordered minerals are often more positively associated with soil C storage^12,49,50^, but it also renders them more susceptible to disruption than their more crystalline counterparts. Depending on mineral properties and soil pH, mineral phases can have positively, neutral, or negatively charged surface sites^36,38^. Minerals can bind OM of opposing charge via sorption, with the strength of the OM–mineral bond influenced by the mineral surface charge. Finally, the susceptibility to dissolution also depends on the chemical stability of the mineral phase, which is controlled by the charge density and redox sensitivity of the metal ions forming the crystal lattice, as well as the covalent or ionic character of the bonds between them^39,51^. In general, the presence of low charge density Ca ions in combination with relatively weak, ionic Ca–O bonds renders minerals like calcite susceptible to mineral dissolution. In mineral phases with stronger covalent character, the presence of redox-active coordinating metals (e.g., in Fe or Mn oxides) may render minerals more susceptible to dissolution. Finally, minerals dominated by strongly covalent, redox-insensitive Si–O bonds (e.g., in phyllosilicates) are less susceptible to dissolution. It follows that mineral phases in MOAs with a low degree of order, high surface charge, and low chemical stability (e.g., ferrihydrite and Al(OH)_3_) are generally more vulnerable to disruption in the rhizosphere; conversely, highly crystalline phases with lower surface charge and high chemical stability (e.g., 1:1 phyllosilicates and hematite) are less vulnerable.

#### Organic matter–mineral interactions

In addition to the characteristics of the OM and mineral phases, MOA vulnerability is also a product of their interactions. The OM–mineral interaction can be characterized by the bond strength between OM and mineral phases, the number of bonds between OM molecules and mineral surface (i.e., denticity), the extent of OM layering on the mineral surface, and the degree of physical entrapment arising from co-precipitation or absorption (Fig. 2c). The potential for OM desorption from minerals depends on the strength of the chemical bond formed between OM and minerals. Here we distinguish (i) inner-sphere complexes characterized by covalent bonds (i.e., ligand exchange); (ii) outer-sphere complexes formed through electrostatic interactions (such as cation or water bridging); and (iii) hydrophobic interactions (e.g., hydrophobic effects and van der Waals forces), with the bond strength decreasing in that order^52,53^.

In addition to the strength of the bond, the number of contact points between OM molecules and mineral surfaces may affect desorption rates, and thus the potential for MOA disruption. For example, the multiple (polydentate) bonds that higher-molecular-weight or polymer organic compounds may form with mineral surfaces lead to lower desorption rates than monodentate bonds formed by, for example, monomers. Moreover, OM has been found to self-assemble into “multilayers”^41,54–57^ or “organo-organic interfaces”^57^ on mineral surfaces, presumably held together by electrostatic or hydrophobic interactions between OM constituents themselves or with metal ions acting as bridging cations. While there is ongoing debate about the conceptualization of OM self-assembly on mineral surfaces^41,56^, it is often invoked that OM in outer layers may be more vulnerable to desorption or other forms of disruption.

And, lastly, the degree of physical entrapment of OM within MOAs may regulate its susceptibility to disruption. On exposed and highly crystalline mineral phases, MOAs may form as simple OM adsorption complexes on the mineral surface. Consequently, parts of OM that are not directly involved in the adsorption complex may remain  relatively susceptible to potential disruption mechanisms. But in many other cases, OM may be physically protected from such disruption mechanisms because it diffuses or is absorbed into nanopores within the mineral phase (e.g., Fe oxides or phyllosilicate interlayers)^58–60^, or because it co-precipitated within a newly formed mineral phase (e.g., Fe and Al oxides)^43,61^.

### A new conceptual framework for MOA vulnerability to disruption

**Fig. 3:**
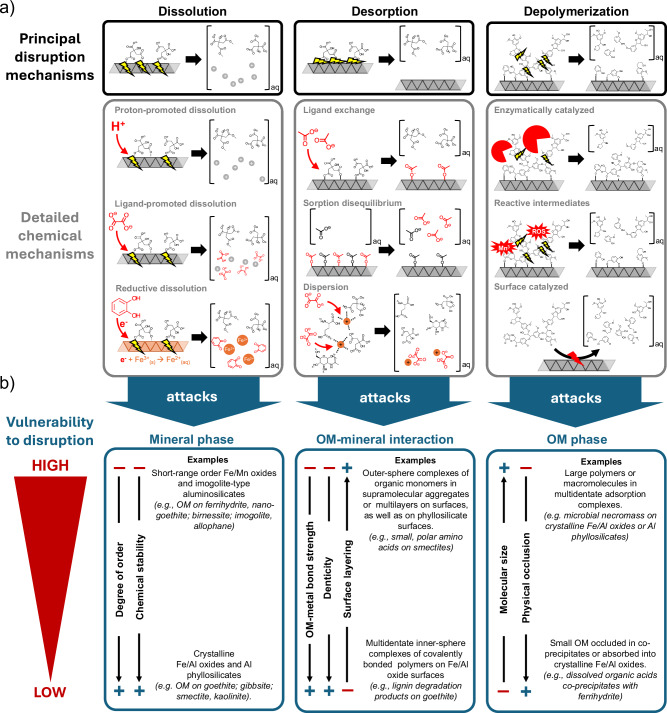
Principal mechanisms disrupting mineral-organic associations (MOAs), their preferred targets, and possible implications for vulnerability. **a** Principal mechanisms of MOA disruption: dissolution, desorption, and depolymerization, with detailed chemical reactions presented below. **b** These mechanisms preferentially attack the mineral phase, organic matter (OM)–mineral interactions, and the OM phase, respectively. Based on the most important MOA characteristics defined in Fig. 2, we present examples of specific MOAs with high and low susceptibilities to each disruption mechanism. Gray mineral phases denote generic mineral surfaces, while ROS denotes reactive oxygen species.

MOA vulnerability to disruption in the rhizosphere depends on the underlying chemical mechanisms at play. The principal mechanisms can broadly be grouped into (a) chemical dissolution of the mineral phase, (b) desorption of OM from the mineral phase, and (c) depolymerization of mineral-bound OM (Fig. 3). These disruption mechanisms target, respectively, the mineral phase, bonds between OM and the mineral phase, or OM itself. In the following, we introduce each MOA disruption mechanism and discuss the inherent vulnerabilities of different MOAs to these mechanisms.

#### Dissolution

The ability of roots and associated microbes to dissolve minerals is well-documented and generally attributed to nutrient acquisition, for example, as roots liberate P from Fe and Al oxides in highly weather soils^21^. But this biologically driven chemical dissolution of minerals in the rhizosphere may also represent a major MOA disruption mechanism^20,25,62^. In soils, we can distinguish between (i) proton-promoted, (ii) ligand-promoted, and (iii) reductive dissolution reactions (Fig. 3a). All three dissolution mechanisms have in common that an external reactant (protons, ligands, or reductants) initially adsorbs to the mineral phase, weakening bonds within the mineral phase and thereby accelerating the release of metal ions into the soil solution^63^. Consequently, individual parts or even the entire mineral phase are dissolved, and any MAOM is mobilized. *Proton-promoted dissolution* occurs when increased proton concentrations in the soil solution (i.e., decreasing soil pH due to proton release by roots) result in proton adsorption to mineral surfaces^32,64^. Once adsorbed, the protonation of surface oxygen (O) atoms weakens metal-O bonds (e.g., Si–O in silicates or Fe–O in oxides), destabilizing the mineral structure and increasing the release of metal ions into solution.

*Ligand-promoted dissolution* is caused by the release of strong ligands (e.g., organic acids) that adsorb to the metal ions in mineral phases. The resulting adsorption complex weakens the other bonds of the complexed ion within the crystal lattice, facilitating the metal ion’s dissolution into the surrounding soil solution^63^. *Reductive dissolution* occurs in the presence of ligands acting as reducing agents (i.e., reductants) that transfer electrons to mineral surfaces^63^. This is principally the case for Fe(III) and Mn(IV) oxides, which accept electrons and become chemically reduced to Fe(II) and Mn(II). Fe and Mn are more soluble in their lower oxidation state (i.e., Fe(II) and Mn(II)), so their reduction renders Fe- and Mn-bearing minerals more susceptible to dissolution. Organic reductants such as organic acids, quinones, or phenols are frequently released in the rhizosphere by roots and associated microbes^3,4,65^ and are capable of reductively dissolving mineral phases and mobilizing MAOM^20,62^. However, it has to be noted that only redox-active Fe and Mn oxides are vulnerable to reductive dissolution^66–68^.

The susceptibility of a mineral phase to dissolution principally depends on the chemical energy and degree of order of the crystal lattice^39,63^. Minerals dominated by highly covalent, non-redox active Si–O bonds (e.g., phyllosilicates) are more difficult to dissolve than minerals dominated by more redox-active bonds (e.g., Fe–O or Mn–O) or ionic bonds (e.g., Ca–O and Al–O). In the presence of high proton, ligand or reductant concentrations, Fe-, Mn-, or Al-rich mineral phases (e.g., Fe oxides and imogolite-type phases) are therefore more susceptible to dissolution than those consisting of relatively Si-rich phases (e.g., phyllosilicates). Moreover, because atoms in mineral phases with a greater degree of order are arranged in a repetitive pattern that minimizes the internal energy, they tend to be energetically less susceptible to dissolution^39,63^. The dissolution of mineral phases with a low degree of order is also favored kinetically, as their inherently larger surface area facilitates the adsorption of protons, ligands, or reductants, thereby accelerating the subsequent dissolution reaction. Consequently, both energetics and kinetics render oligomers, short-range order phases of Fe-, Mn-, and Al-oxides, and imogolite-type materials particularly vulnerable to dissolution in the rhizosphere^18,20,25,28,62,69–72^. It is important to note, however, that the dissolution of mineral phases is frequently slowed or inhibited when adsorbed or co-precipitated OM blocks the reactive sites for protons, ligands, or reductants to attack^73,74^.

#### Desorption

Desorption of MAOM occurs if chemical bonds between OM and mineral phases are broken, liberating previously bound OM into the soil solution^19,20^. In principle, OM bound through all types of sorptive interactions can be subject to desorption. But the likelihood that OM will be desorbed from mineral surfaces decreases with increasing strength of the OM-mineral bond (Fig. 2c). In general terms, we distinguish three types of desorption mechanisms: (i) ligand exchange reactions, (ii) sorption disequilibrium, and (iii) chemical dispersion (Fig. 3a).

*Ligand exchange* occurs when OM with relatively high affinity for mineral surfaces replaces OM with lower affinity. This exchange arises when the interaction between the newly adsorbed ligand and the mineral surface is more thermodynamically favorable than the interaction between the previously adsorbed ligand and the mineral phase. That is why, at the soil profile scale, dissolved organic matter (DOM) percolating through soil can exchange between 15-32 % of adsorbed OM on minerals^75^. In the rhizosphere, these reactions can occur when roots or microbes increase concentrations of organic (e.g., organic acids) or inorganic (e.g., PO_4_^3−^, Al^3+^, H^+^, OH^−^) ions^18,19,76^. Consequently, OM bound by relatively weak outer-sphere complexes is particularly vulnerable to ligand exchange, for example, via relatively weak organic acids released by roots and microbes^18^. Similarly, many inorganic ions can, when present at elevated concentrations, exchange OM held in outer-sphere complexes on phyllosilicate surfaces due to increases in ionic strength^77^. But it is important to note that ligand exchange with strong complexing agents (e.g., strong organic acids or siderophores) is the only mechanism that effectively desorbs OM bound via strong, inner-sphere complexes commonly found in MOAs formed by Fe and Al oxides, representing a critical disruption mechanism for MOAs that are commonly perceived as the most stable.

Sorption disequilibrium arises when DOM concentrations change due to external factors, as may be the case when microbes consume DOM in the rhizosphere. If DOM concentration decreases, the soil solution may become undersaturated in DOM relative to MAOM. The resulting sorption disequilibrium favors desorption of OM from minerals over adsorption, mobilizing MAOM as DOM until a new equilibrium is established^53,78,79^. A decrease in DOM concentrations in rhizosphere pore water could consequently cause desorption, disrupting MOAs and making organic molecules more bioavailable. For example, reactive transport modeling of rhizosphere sorption dynamics recently suggested that diel cycles in microbial activity sufficiently draw down DOM concentrations to result in the desorption of organic acids bound to Fe oxides via outer-sphere complexes^33^. This further affirms the idea that weakly bound MAOM is released first into solution^80^.

*Chemical dispersion* describes the process by which supramolecular assemblies of mineral phases and OM, formed by non-covalent interactions, may be mobilized into the soil solution through the action of chemical agents. Such dispersing agents (e.g., ionic or non-ionic surfactants) increase the repulsive forces between mineral phases and OM. Dispersion can act on non-covalent bonds between OM and monomeric metal phases (e.g., Al, Fe, or Ca ions) in amorphous supramolecular aggregates^81,82^ or multi-layers on mineral surfaces^54^. For example, introducing organic acids^83,84^ or increasing pH^81^ effectively disperses supramolecular OM aggregates into individual organic molecules. Dispersion in these cases can be attributed to organic acids competing with OM for “bridging” metal ions within supramolecular OM aggregates or increased OM protonation, enhancing repulsive forces among organic molecules.

#### Depolymerization

MOAs can also be disrupted by depolymerization reactions catalyzed by: (i) enzymes (ii) reactive intermediates of redox cycling, or (iii) mineral surfaces in the rhizosphere (Fig. 3a). Depolymerization reactions may cleave covalent bonds in larger covalent polymeric MAOM, destabilizing and sometimes also mobilizing the resulting smaller products. Such depolymerization reactions may lead to a partial release of OM from MOAs, directly targeting MAOM without requiring dissolution of or desorption from the mineral. Wang et al.^23^ demonstrated that ectomycorrhizal fungi release extracellular enzymes which attack mineral-bound proteins by cleaving oligomeric or monomeric moieties. Depending on the type of MOA, however, steric effects may inhibit interactions between the enzyme’s active site and MAOM, or the enzyme may become deactivated upon contact with the mineral surface^24^. It is thus likely that MOAs containing large polymeric compounds with high surface coverage, as well as OM adsorbed to surfaces via layers or stacks extending away from the surfaces, are more vulnerable to enzymatic depolymerization than smaller compounds directly bound to the surface or OM entrapped in coprecipitates (Fig. 3b).

Depolymerization reactions can also be mediated abiotically through minerals and reactive intermediates. For example, the surfaces of Mn and Fe oxides can act as catalysts, partially oxidizing OM upon contact^38,64,68^. Fe redox cycling in the rhizosphere^32^ can also produce reactive intermediates such as small, diffusible reactive oxygen species^85,86^. Reactive oxygen species like peroxide and hydroxyl radicals have the potential to oxidize MAOM. However, the role of reactive oxygen species production in the rhizosphere in disrupting MOAs remains underexplored.

In summary, MOA disruption mechanisms directly target bonds within the mineral phase, the OM, or the interaction between both components. The efficacy of a specific disruption mechanism depends on the inherent physical and chemical characteristics of the targeted MOA component. Based on the information presented above, we can place specific MOAs on a vulnerability spectrum (Fig. 3b). To date, this information is mostly based on very simple model systems—often batch experiments with pure minerals and OM subjected to different conditions (model exudates, pH or E_h_ changes, etc.) to favor dissolution or desorption—that do not include plant, their roots, and associated microbes. Our proposed MOA vulnerability spectrum (Fig. 3), therefore, provides a hypothetical framework to guide novel research that explicitly assesses the importance of MOA disruption mechanisms in real plant–soil systems.

### Triggers of MOA disruption in the rhizosphere

**Fig. 4:**
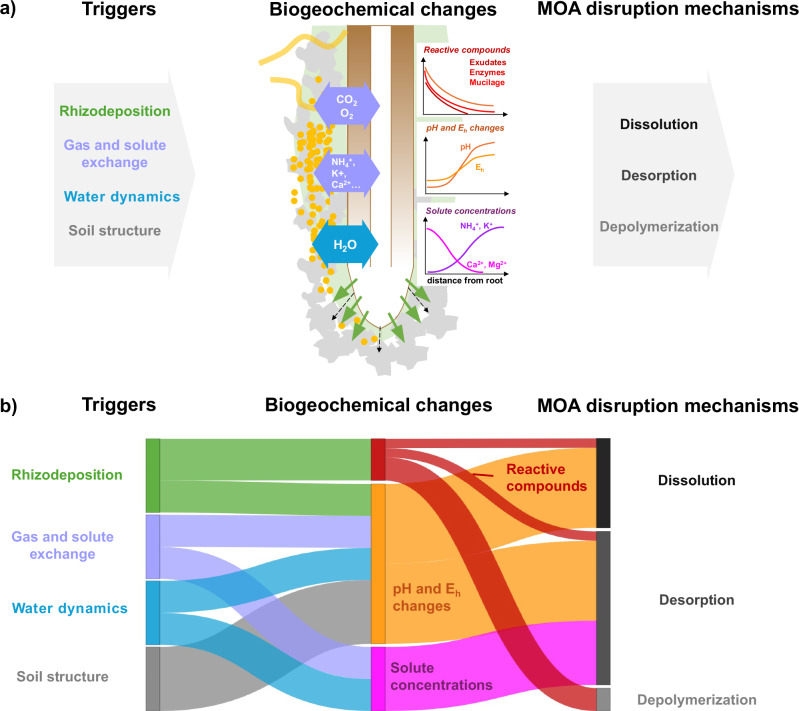
Rhizosphere processes and their effects on mineral-organic associations (MOAs) disruption in the rhizosphere. Panel **a** shows how interactions and feedback between plant root growth and associated microbial activity promote changes in rhizodeposition, gas and solute exchange, water dynamics, and soil structure. These triggers result in changes in the abundance of reactive compounds (e.g., exudates, siderophores, mucilage, and enzymes), pH and redox (*E*_h_) conditions, and solute concentrations. These biogeochemical changes can stimulate specific MOA disruption mechanisms. Panel **b** qualitatively links specific rhizosphere triggers and biogeochemical changes to specific MOA disruption mechanisms, highlighting that each rhizosphere trigger can cause the disruption of MOA in a wide variety of ways. The triggers may interact and even act synergistically, adding another level of complexity. For instance, rhizodeposition may enhance or reduce water flow, affecting gas and solute exchange.

While there is a significant body of literature on the chemical mechanisms that might disrupt MOAs, much less is known about the rhizosphere processes that may trigger such MOA disruption mechanisms (Fig. 4). Dynamic interactions between root and associated microbial activity in the rhizosphere drive rhizodeposition, gas and solute exchange, water fluxes, and modifications of soil structure^3,4,27,29,35,87,88^. These rhizosphere processes may serve as triggers promoting MOA disruption mechanisms via various biogeochemical changes, such as changes in soil pH, redox status, and solute concentrations.

#### Reactive organic compounds

Rhizodeposition releases small reactive compounds that *directly* facilitate MOA dissolution and desorption mechanisms (Fig. 3)^19,20,62^. Among these compounds are *simple carboxylates* (predominantly aliphatic or aromatic acids), which complex and dissolve metal oxides and potentially disrupt MOAs via ligand-driven dissolution^89,90^. Ligand concentrations and their molecular properties (e.g., size, charge and electronic properties) influence their reactivity towards metal ions, and thus their potential to promote dissolution or desorption. Roots and microbes also release *siderophores*—organic compounds with a high ability to complex and dissolve metals—to scavenge nutrients^91^. While the primary function of siderophores is Fe acquisition, their structural diversity confers variable affinity for different metals^92,93^. They can dissolve minerals independently or synergistically in tandem with other organic ligands^74,94–96^. In addition, roots and associated microbes release a variety of *reductants*—phenols, organic acids, and aromatic compounds acting as electron donors to minerals— which can reductively dissolve Fe and Mn oxides (see reductive dissolution; Fig. 3)^3,18,20,97^. Furthermore, roots and microbes release a variety of hydrolytic and oxidative *extracellular enzymes* capable of depolymerizing complex OM, which may contribute to the dissolution of smaller fragments from MAOM that render them more accessible (see enzymatic depolymerization; Fig. 3)^24^. While small reactive compounds may *directly* trigger dissolution, desorption, or depolymerization reactions that disrupt MOAs, less reactive compounds (e.g., sugars or amino acids) may also mediate microbial processes that disrupt MOAs *indirectly*. For example, the release of simple sugars and amino acids may promote the microbial production of more reactive organic ligands or enzymes, which then, in turn, accelerate mineral dissolution or depolymerization^17,18^. Larger rhizodeposits, such as mucilage and other extracellular polymeric substances, have also been found to have a significant impact on microbial activity, but their effects on MOA disruption are less clear.

#### pH and redox changes

Rhizodeposition, gas or solute exchange, water dynamics and modification of the pore system may alter the proton balance of the rhizosphere, leading to *pH changes* that may undermine MOA stability. Root activity routinely modifies soil pH by more than a full unit through the release of H^+^ and OH^−^ ions, organic acids, and CO_2_ that builds up and forms carbonic acid^98–100^. Root-driven acidification facilitates proton-promoted dissolution in the rhizosphere (Fig. 3)^101,102^. Although acidification appears to be more common than alkalinization of the rhizosphere, both the direction and magnitude of pH change are highly context-dependent. For example, Blossfeld et al.^100^ found that wheat roots acidified soil at the root tip but alkalized other parts of the rhizosphere. This rhizosphere alkanization can promote MOA disruption via dispersion (Fig. 3).

The availability of reactive organic compounds, along with gas and water dynamics and changes in pore connectivity, may also alter *redox potential (E*_*h*_*)* in the rhizosphere, a principal control on the solubility of reducible metal oxide minerals^103^. Root-derived organic substrates can fuel microbial respiration, which, along with cellular respiration within the root, can cause significant O_2_ depletion in the rhizosphere^30,33,104^. In addition, root elongation and expansion compacts surrounding soil^105–109^ and dramatically decreases pore sizes^27^, slowing O_2_ diffusion. Moreover, mucilage formation^3,29^ maintains higher soil moisture content in the rhizosphere even under strong negative water potentials (e.g., when the root is actively taking up water)^110–112^. As a result of increased O_2_ consumption and diminished supply (due to compaction and higher water content) in the rhizosphere, localized anoxic microsites can emerge^28,30^, lowering *E*_*h*_ and driving microbes to utilize energetically less favorable alternative electron acceptors such as Fe and Mn oxides for respiration. Consequently, microbes reductively dissolve Fe and Mn oxides (Fig. 3)^68,113^, potentially disrupting MOAs in the process^25^. Despite their clear potential, the impacts of pH and E_h_ changes in the rhizosphere on MOA disruption have rarely been explored.

#### Solute concentration

Rhizodeposition, water dynamics, and nutrient acquisition in the rhizosphere can significantly alter solute concentrations and thus MOA stability. Plant transpiration and hydraulic redistribution drive water fluxes to and from the root^114^. These water fluxes carry solutes towards or away from the rhizosphere. For example, rhizosphere modeling suggests that diel variations in plant water and nutrient uptake result in daytime accumulation of low-demand cations (e.g., Ca^2+^, Mg^2+^, and Na^+^), which were shown to competitively desorb (via exchange reactions) high-demand cations such as NH_4_^+^ and K^+^ from rhizosphere soil^115^. Diel changes in rhizosphere cation concentrations could disrupt MOAs directly (e.g., through ligand exchange) or by altering the vulnerability of MOAs to disruption (e.g., by influencing the bond strength within the MOA that remains after cation exchange has occurred). Similarly, diel variations in the release or consumption of organic compounds from roots can either oversaturate or undersaturate the soil solution in DOM relative to MAOM, causing sorption disequilibria that favor sorption or desorption (Fig. 3). Garcia Arredondo et al.^33^ showed that increased root-derived DOM during the day favored sorption, while enhanced microbial DOC consumption at night favored desorption. These modeling studies suggest a clear potential for dynamic exchange reactions or dynamic changes in sorption equilibria in the rhizosphere that favor MOA disruption, but no experimental evidence exists to date.

### Vulnerability and dynamics of MOA in the rhizosphere—research priorities

Our critical review of the literature identifies known and potential drivers, mechanisms, and controls of MOA disruption in the rhizosphere. We have demonstrated how MOA vulnerability within a given ecosystem can be equally influenced by the inherent properties of MOAs and the nature and efficiency of rhizosphere processes that stimulate disruption. The resulting spectrum of vulnerability provides a conceptual basis and hypothetical framework for identifying key research priorities. We suggest that future research should prioritize elucidation of specific MOA disruption mechanisms, their ecosystem-level controls and specificity, and their response to shifts in environmental conditions. To make the necessary progress on all of these critical research fronts, multi-pronged approaches are needed that integrate reductionist model system studies, well-controlled experiments with real plant-soil systems, and process-level modeling. Understanding what (plant or edaphic) factors control rates of MOA disruption in a given ecosystem will put us in a better position to project the balance of MOA disruption and formation to global scales.

#### Can we systematically link MOA characteristics to potential vulnerability?

Our proposed vulnerability spectrum strongly suggests that distinct types of MOA have different susceptibilities to specific disruption mechanisms. This observation implies that efforts to study MAOM destabilization in the rhizosphere require pairing conventional quantification of MAOM pools (e.g., by physical fractionation) with a more nuanced analysis of the MOA characteristics that determine potential vulnerability to disruption. Each soil contains a significant diversity of MOA types, with the predominant types varying dramatically by soil ecosystem^36,50^. Selective dissolution^116^, spectroscopic^117^, and mass spectrometric^118,119^ approaches are increasingly available and high-throughput, making a comprehensive MOA characterization (i.e., mineral type, OM functionality, and their interactions) across soil ecosystems more feasible. At the same time, studies assessing MOA (and, by association, MAOM) vulnerability to specific disruption mechanisms^18,23^ or environmental changes^120^ are becoming more common. Combining such detailed information on MOA characteristics and vulnerabilities—as pioneered, for example, by syntheses linking mineralogical data^50,121^ to soil C vulnerability^122,123^—represents a critical step toward global-scale projections of MOA vulnerability.

#### Beyond rhizodeposition: what mechanisms disrupt MOAs in the rhizosphere?

Our understanding of the potential for MOA disruption in the rhizosphere is limited by the nearly exclusive focus on the effects of simple reactive compounds released by roots or associated microbes. Relevant mechanistic questions remain regarding the relative importance of plant-released ligands that directly attack MOAs compared to the release of soluble compounds that indirectly attack MOAs via the production of microbial secondary metabolites or exo-enzymes^17,18,23,24^. This review illustrates that some MOAs are more vulnerable to attack by such reactive compounds, while others are less. Even in the absence of strong attack by reactive compounds, MOAs may still be vulnerable to complex plant-microbial-soil interactions that alter rhizosphere conditions like *E*_*h*_, pH, water dynamics, and solute transport. To fully assess the potential for MOA disruption in the rhizosphere, more studies are needed that explicitly address effects of emergent anoxic microsites^124^, low pH zones^30,33^, wetting and drying effects^110,114,125^, and sorption disequilibria^126^. It remains to be seen whether plant roots themselves or associated microbes trigger specific MOA disruption pathways and mechanisms, and to what extent they act synergistically.

#### Where and when does MOA disruption occur in the rhizosphere?

Root and associated microbial activity that might trigger MOA disruption mechanisms are highly dynamic in space and time. For example, if we consider rhizodeposition as a trigger of MOA disruption, it becomes evident that both the amount and type of rhizodeposits vary spatially (from single roots to ecosystem scale) and temporally (diel cycles to successional stages). Single roots have well-defined developmental stages, ranging from the root tip to the zones of elongation and suberized zones, each with distinct rhizodeposition patterns^21,104,127^. In each zone, we expect variations in quality and quantity of reactive compounds (ligands, enzymes, or mucilage), which may translate to differences in MOA disruption pathways and efficacies. Additionally, diel photosynthesis cycles cause spikes in rhizodeposition and associated microbial activity during the day^29,128,129^. Moreover, at the scale of the entire root network, rhizodeposition hotspots and hot moments emerge across phenological stages^130–133^, seasons^134^, and within different locations in the root network^135^. At the ecosystem level, rhizodeposition is expected to vary not only quantitatively with root density and depth^136^, but also with root functional traits (e.g., nutrient versus water acquisition)^137^.

The spatiotemporal variability in rhizodeposition is mirrored by observations of mineral transformations at complementary scales. For example, we observe differential mineral transformation in the rhizosphere along single growing roots^30^, over diel cycles^33,115^, during seasonal root growth and senescence^138,139^, and over pedogenic time scales^118^. Such observed patterns of root activity, associated microbial dynamics, and mineral transformations provide a clear indication for the potential of MOA disruption, but also suggest that it is extremely variable in space and time across different ecosystems. These considerations highlight that future efforts to quantify MOA disruption rates (in relation to MOA formation rates) need to consider potential hotspots and hot moments of MOA disruption within a given ecosystem.

#### Which MOA disruption mechanisms dominate across different soil ecosystems?

If MOA characteristics are known, one can postulate which MOA disruption mechanisms may be the most prevalent across different ecosystems. For example, mechanistic considerations suggest that mineral dissolution and ligand exchange reactions are the most effective MOA disruption mechanisms for short-range order oxides and metal–organic complexes, respectively. Because humid soil systems (temperate and tropical) are often dominated by oxides, plants and associated microbes have developed nutrient scavenging strategies tailored to target these minerals^21^. For instance, ectomycorrhizal fungi dominating temperate and boreal forests show a strong potential for mineral weathering through the production of organic acids and ligands^140,141^. In tropical systems, plant roots and associated fungi rely on specialized P acquisition strategies that rely on organic acids and siderophores^142^. Additionally, rhizodeposition can create O_2_ limitations in the rhizosphere, which are known to cause reductive dissolution of minerals in humid tropical^143,144^, temperate grasslands^118^, or agricultural systems^145^. These observations suggest that plant root-induced MOA disruption in these systems relies on specialized mechanisms involving ligand-driven or reductive dissolution. *Desorption* of relatively weakly held outer-sphere complexes via exchange reactions and disequilibrium is particularly important for phyllosilicates or amorphous metal-organic complexes. Thus, MOA disruption via desorption of outer-sphere complexes may dominate in soils rich in phyllosilicates, such as those in arid- or semi-arid regions^50^, or in soils dominated by Ca–organic complexes, such as relatively young alkaline soils^146^. Additionally, *dispersion* is an effective mechanism for disrupting MOAs consisting of supramolecular structures, whether coordinated with monomeric metals or not. These structures may be found in Al-rich volcanic^60^, Ca-rich alpine^146^, or temperate grassland soils^147^.

Lastly, we posit that *depolymerization* reactions may be most effective for the destabilization of complex polymeric compounds bound to crystalline minerals (oxides and phyllosilicates). This is potentially the case in grassland or agricultural soils where particulate or polymeric plant or microbial biomass residues are intimately associated with crystalline minerals^55,148^. This hypothesis further agrees with the observation that grassland and agricultural systems are dominated by arbuscular mycorrhiza, which are generally considered to rely much less on direct nutrient mining strategies than ectomycorrhiza^149,150^. In contrast to ectomycorrhizal fungi, arbuscular mycorrhizal fungi do not possess a large repertoire of reactive ligands or enzymes to weather minerals or acquire nutrients. But arbuscular mycorrhizae are known to stimulate the activity of saprotrophs in the rhizosphere environment through the release of simple organic compounds, enhancing OM decomposition and nutrient availability^151–153^. It seems feasible that arbuscular mycorrhiza may trigger *indirect* pathways, via secondary microbial metabolites or enzymes, that could promote depolymerization of complex, polymeric compounds in grassland systems dominated by phyllosilicates.

These considerations suggest that, depending on the types of MOA dominating within a given soil ecosystem, plants and associated microbes may have adapted to employ specific mechanisms to facilitate the disruption of MOA. This hypothetical framework for MOA vulnerability across ecosystems warrants testing, and we propose that future research should validate if and to what extent MOA, plant, and microbial traits predict the prevalence and rates of specific MOA disruption mechanisms within a given soil.

#### To what extent is MOA disruption in the rhizosphere tied to ecosystem nutrient cycling?

We established the potential vulnerability of MOAs to rhizosphere processes across different ecosystems, prompting the question: do plants benefit directly from MOA disruption, or is it simply an inadvertent outcome of rhizosphere dynamics? MOAs are rich in organic nutrients, often representing the largest pools of N and P^17,154^ in soils. Historically, this mineral-bound organic N and P pool has been considered relatively inaccessible to plants and microbes. The evidence presented here and elsewhere^17^ indicates that plant and microbial MOA disruption increases the availability of organic N and P pools, as well as a variety of other micronutrients contained within MAOM. If MOA disruption enhances nutrient availability and recycling, one could expect an overall increase in plant and microbial productivity. Such positive feedback might destabilize MAOM, but could also enhance nutrient recycling and ecosystem productivity, potentially leading to greater C inputs belowground. To what extent MOA disruption rates and mechanisms depend on MOA nutrient availability and biological nutrient demand, thus warrants future research.

#### What factors govern the balance between MOA formation and disruption?

Theoretical and experimental evidence^19,20,25^ indicates that rhizosphere processes promote both MOA formation and disruption. Root biomass and turnover in soils can be predicted based on selected ecosystem properties^155,156^. Based on current conceptual frameworks that emphasize the importance and prevalence of root-derived C in MOAs^157^, one could assume that root biomass and turnover within an ecosystem are proportional to potential MOA formation. If one then assumes that MOA disruption rates generally also scale with root biomass and turnover as well, one might postulate that both MOA formation and disruption follow broadly similar patterns. However, it is unlikely that both MOA formation and disruption are synchronous in space and time. It is much more likely that they are asynchronous, and it will be important to elucidate when and where MOA formation or disruption prevails.

Is the influence of roots on the balance of MOA formation and disruption in specific ecosystems perhaps modulated by plant, microbial or soil characteristics? The increasing availability of global datasets on root biomass and turnover^155,156^, soil mineral composition^50^ and C turnover times^116,158^ provides rich opportunities to explore the modulating effect of mineral reactivity on this balance. The balance between MOA formation and disruption may also be regulated by ecosystem-specific plant traits. For instance, fast-growing plant species with strong nutrient scavenging traits may favor disruption over formation. Similarly, soil nutrient limitations (e.g., of N and P) cause an increase in the release of reactive compounds that may promote MOA disruption through direct chemical or indirect, microbially mediated pathways^17,18,25^. Lastly, inoculation with specific microbial symbionts (e.g., mycorrhizal fungi) is currently being encouraged as a strategy to promote crop yield and soil C sequestration in agricultural systems^159,160^. The resulting new fungal necromass may contribute to the stabilization of C in MOAs^161^, and increased fungal activity may also enhance the capacity to mobilize or dissolve mineral nutrients, supporting increased crop yield, but potentially also disrupting MOAs in the process. In the future, it will be critical to determine how such complex plant-microbe-mineral interactions shape the efficacy with which MOAs are disrupted, and how this relates to the efficacy with which new MOAs are formed.

#### How does the balance between MOA formation and disruption respond to global change?

How global change factors impact soil C stocks is a key scientific challenge in ecosystem science. While there has been extensive research on soil C loss in response to climate and land use change, rhizosphere studies have largely focused on estimating climate change impacts on root C inputs^162^ and associated priming^163^. For example, climate change factors such as elevated soil temperature^164^, increased CO_2_^165^, or extreme drought conditions^166,167^ can alter plant community composition or physiology in ways that alter rhizodeposition^168,169^. While there have been a few studies that directly address how such changes in rhizodeposition affect MOA formation^25,157,170^, very little is known about the impacts on MOA disruption^25^. Given the diversity of MOA disruption mechanisms potentially operating in the rhizosphere, it seems likely that the prevalence of disruption mechanisms will be affected by not just changes in rhizodeposition (quantity, composition, etc.)^168,169^, but also other adaptations of the plant-soil system to changes in climate (e.g., water availability or prevalence of pathogens) and land use (e.g., fertilization, tillage, compaction, and crop selection). We thus advocate for a broad suite of future studies that assess climate and land use effects on the balance of MOA formation and disruption in the rhizosphere.

### Shifting paradigms: toward a new focus on the dynamic nature of MOAs

Significant research efforts are currently underway to provide policymakers and land managers with knowledge to improve soil C storage, for example by promoting stabilization of root C inputs. Frequently, these efforts are based on the premise that promoting plant root growth will stabilize associated C inputs within newly formed MOAs in the rhizosphere. Here, we document that plant roots and associated microbes have the potential to promote MOA disruption in the rhizosphere through a variety of biogeochemical pathways and mechanisms, potentially destabilizing MAOM and causing soil C loss. Based on this evidence, we postulate that (i) dynamic rhizosphere processes, beyond rhizodeposition, play an important role in MOA disruption; (ii) the efficacy of these MOA disruption mechanisms in different soil ecosystems depends on the interaction between rhizosphere processes and MOA characteristics; and (iii) plant-microbe-soil feedbacks induced by climate change may alter MOA disruption rates. These considerations highlight that accurate predictions of soil C dynamics must account for the dynamic nature of MOAs in the rhizosphere, explicitly considering both MOA formation and disruption. Determining this balance between MOA formation and disruption in the rhizosphere—and its consequences for rates of MAOM stabilization and destabilization—is thus critical to efforts to improve soil C storage and better predict the impacts of climate and land use change. Addressing this scientific challenge requires a paradigm shift and a new focus on the dynamic nature of MOAs.
